# Global Research Trends in Pediatric Trauma From 1968 to 2021: A Bibliometric Analysis

**DOI:** 10.3389/fped.2021.762531

**Published:** 2021-10-28

**Authors:** Tauseef Ahmad, Linlin Hua, Muhammad Khan, Ghulam Nabi, Suliman Khan, İlgün Özen Çinar, Haroon, Sajid Jalal, Mukhtiar Baig, Hui Jin, Xiaoyan Wang

**Affiliations:** ^1^Department of Epidemiology and Health Statistics, School of Public Health, Southeast University, Nanjing, China; ^2^Key Laboratory of Environmental Medicine Engineering, Ministry of Education, School of Public Health, Southeast University, Nanjing, China; ^3^Department of Advanced Medical Research, The Second Affiliated Hospital of Zhengzhou University, Zhengzhou, China; ^4^Department of Biotechnology and Genetic Engineering, Hazara University Mansehra, Mansehra, Pakistan; ^5^Key Laboratory of Animal Physiology, Biochemistry and Molecular Biology of Hebei Province, College of Life Sciences, Hebei Normal University, Shijiazhuang, China; ^6^Department of Public Health Nursing, Faculty of Health Sciences, Pamukkale University, Denizli, Turkey; ^7^College of Life Science, Northwest University, Xian, China; ^8^Department of Pathophysiology, College of Basic Medical Sciences, Dalian Medical University, Dalian, China; ^9^Department of Clinical Biochemistry, Faculty of Medicine Rabigh, King Abdulaziz University, Jeddah, Saudi Arabia; ^10^Department of Child Healthcare, Hubei Maternal and Children's Hospital, Wuhan, China

**Keywords:** pediatric trauma, bibliometric analysis, web of science, VOSviewer software, global trend

## Abstract

**Introduction:** Every year, millions of children die from preventable causes worldwide. According to World Health Organization, injuries are the leading cause of disability and death among all age groups below 60 years.

**Aim:** This study aimed to evaluate the global research outcomes and trends, and some key bibliometric indicators in pediatric trauma.

**Methods:** A descriptive bibliometric analysis study was designed. On June 14, 2021, an electronic search was performed in the Web of Science Core Collection database using the potential searching keywords “Pediatric AND Trauma” in the title field without any limitations. The search was performed using the Boolean search query method. The data were downloaded in plaintext and comma-separated values format. The required graphs were generated using OriginPro 2018. Furthermore, the data were transferred to HistCite™ software for bibliometric analysis. In addition, the obtained data were plotted for network visualization mapping using VOSviewer software version 1.6.15 for windows.

**Results:** A total of 2,269 documents were included in the final analysis. The included documents were authored by 7,894 authors and published in 395 research and academic journals, mainly in the English language (*n* = 2,222). The main document types were articles (*n* = 1,276, citations = 18,244), and meeting abstracts (*n* = 331, citations = 19). Pediatric (*n* = 2,269) and trauma (*n* = 2,257) were the most widely used keywords. The most productive year was 2019 (*n* = 184, citations = 527). The most prolific author was Upperman JS (*n* = 29, citations = 202). The most attractive journals in pediatric trauma research were *The Journal of Trauma and Acute Care Surgery* (*n* = 290, citations = 5,199) and the *Journal of Pediatric Surgery* (*n* = 256, citations = 5,088). The most active institute was the University of California System (*n* = 110). The most dominant country was the United States of America (USA) (*n* = 1,620, citations = 22,983). The USA and Canada had the highest total link strength, 103 and 70, respectively.

**Conclusion:** This study provides a comprehensive overview of research output in pediatric trauma. The USA continues to dominate scientific research and funding in pediatric trauma. Findings of the current study will help the researchers and clinicians to understand the recent achievements and research frontiers. Collaborative research initiative needs to be established between institutions in developed and developing countries and among researchers.

## Introduction

Pediatric trauma is a potentially fatal injury to children and is the leading cause of death for children in the United States, killing more than all other causes combined (1). These types of injuries necessitate hospitalization and, in most cases, required emergency surgery (2, 3). In 1978, the Advanced Trauma Life Support course (ATLS) was introduced in the United States to standardize trauma assessment in rural settings. This trauma assessment and education standard has spread to over 60 countries. It is also used to improve trauma outcomes. Despite widespread ATLS training, pediatric trauma centers often fail to follow the ATLS protocol (4).

Globally, every year, millions of children die from preventable causes. According to the World Health Organization (WHO) statistics, in 2002, an estimated 875,000 children (age of 18 years) died due to injuries (5). Furthermore, WHO statistics show that injuries are the leading cause of disability and death among all age groups below 60 (6).

Injuries severely affect the welfare and health of the public, regardless of economic status or geographic location, and lead to disability, premature death, lost productivity, and medical costs (Corso et al. (7)). However, significant advancements and development have been made in trauma treatment and prevention. However, inequalities (race and socioeconomic disparities) in health outcomes in the health care system have been uncovered for many conditions. A meta-analysis conducted by Haider et al., reported that the black race had higher odds of death than the white race (OR 1.19 95% CI 1.09–1.31) (8).

Bibliometric indicators are precious tools for assessing research trends, publication frequency, most prolific authors, productive countries, influential studies, and other bibliometric indicators combined with expert knowledge (9). Bibliometric studies are of great interest, provide a comprehensive overview of research trends, and assess the scientific published literature. In recent times, such information is essential for both researchers and clinicians. Bibliometric analysis plays a significant role by providing the referral point to the researchers, policymakers, and practitioners.

Globally, many studies have been conducted on the types, diagnosis, treatment, and prevention of pediatric trauma by the researchers. Bibliometric analysis evaluating in medical research are relatively common in the literature. When the bibliometric analyses on pediatric trauma are examined; such as brain injuries (10), dental injuries (11), anterior cruciate ligament injuries (12), pediatric orthopedic surgery (13), child maltreatment (14) studies have been done. In bibliometric surveys conducted in the field of pediatric trauma in the literature, have evaluated a particular field or subject. However, according to the literature through the Web of Science database, there is no a general bibliometric analysis on pediatric trauma. Therefore, this study was designed to evaluate pediatric trauma research trends and outcomes by determining the leading authors, journals, institutes, countries, and key bibliometric visualization indicators.

## Methods

### Study Design

A descriptive bibliometric analysis study.

### Database and Search Strategy

**Figure 1:**
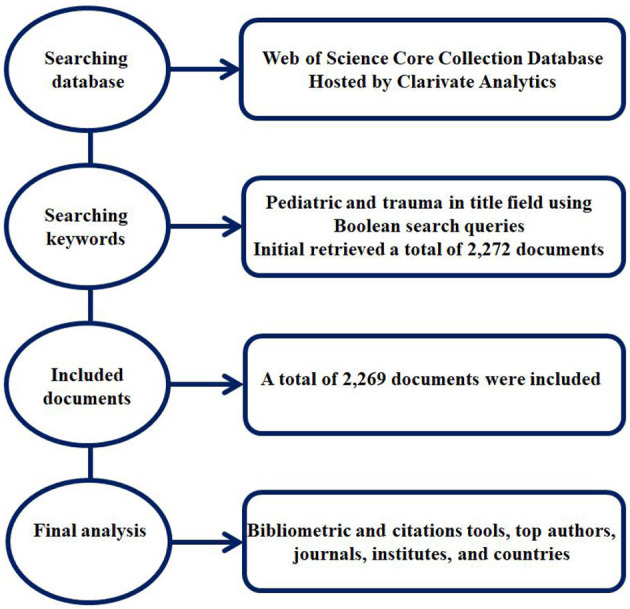
Flow chart of documents used in the final analysis.

The Web of Science Core Collection database hosted by Clarivate Analytics, (https://clarivate.com/webofsciencegroup/solutions/web-of-science/), United States of America (USA) was preferred due to its extensive use in bibliometric research, providing great convenience in obtaining data and analyzing its coverage of a large number of journals in the medical and social sciences, and the screening of a large number of journals. On June 14, 2021, the Web of Science Core Collection database was searched, using the search keywords “Pediatric AND Trauma” in the title field without any limitations, as shown in Figure 1. The search was performed accordingly to Boolean search query method in the search engine. The searching database was accessed using the online library portal of Southeast University, Nanjing, China (http://www.lib.seu.edu.cn/).

### Data Extraction and Analyses

The data were downloaded in plaintext and comma-separated values (CSV) format and then transported into Microsoft Excel 2016 to calculate the frequencies and percentages. The following data were extracted from retrieved documents; author's name, year of publication, journals, funding agencies, institutes, countries, and highly cited papers. The required graphs were generated using OriginPro 2018 for windows (https://www.originlab.com/). Furthermore, the bibliometric analysis was performed through HistCite™ software (http://www.histcite.com/). HistCite™ software easier for individuals to perform bibliometric analysis and visualization tasks (15). In addition, the plaintext dataset was plotted for network visualization mapping (co-authorship countries and author's keywords) through VOSviewer software version 1.6.15 for windows (https://www.vosviewer.com). VOSviewer is widely used in bibliometric analysis, especially evaluates networks among highly cited articles, cartography, and cluster analysis (16).

## Results

### Characteristics of Global Research Output on Pediatric Trauma

The initial search yielded 2,272 documents; after transferring the data into HistCite™ software, 2,269 documents were included in the final analysis. The included documents were authored by 7,894 authors and published in 395 research and academic journals, mainly in the English language (*n* = 2,222, citations = 26,980), as shown in Supplementary Figure 1.

**Figure 2:**
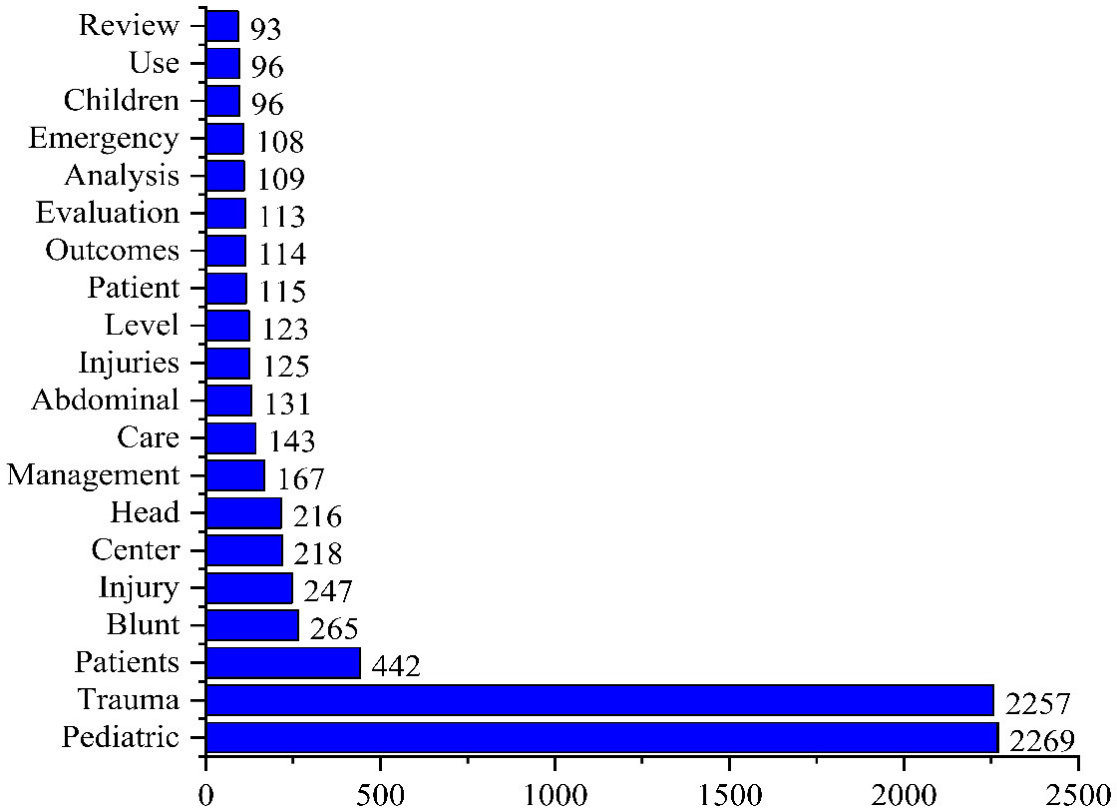
Top 20 most commonly used keywords.

**Figure 3:**
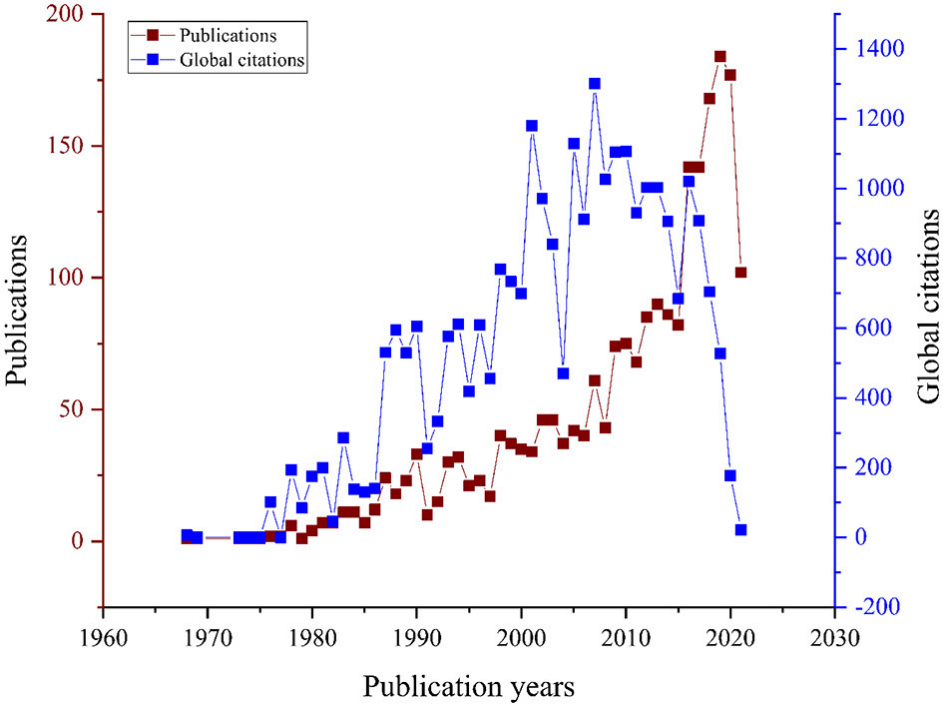
Year of publications and citations of included documents. In this Figure, 12 documents were excluded based on the missing year of publication.

The main document types were articles (*n* = 1,276, citations = 18,244), meeting abstracts (*n* = 331, citations = 19), and proceedings papers (*n* = 308, citations = 6,568) as shown in Supplementary Figure 2. The most commonly used keywords occurrence were pediatric (*n* = 2,269), trauma (*n* = 2,257), and patients (*n* = 442), as shown in Figure 2. The most productive year in publications was 2019 (*n* = 184, citations = 527), while the most cited year was 2007 (*n* = 61, citations = 1,300), as shown in Figure 3.

**Figure 4:**
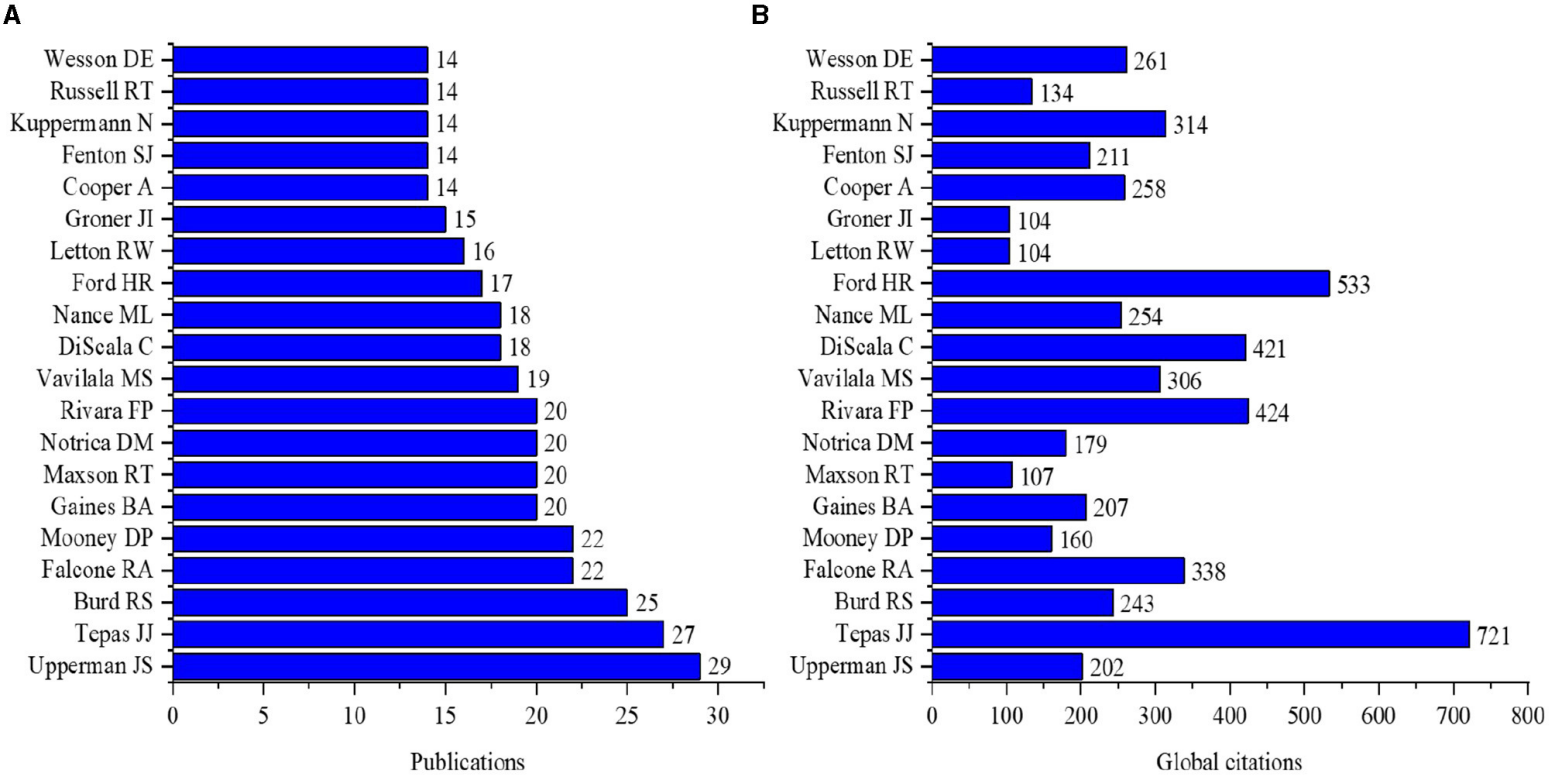
Top 20 authors in pediatric trauma research **(A)** Publications **(B)** Global citations.

**Figure 5:**
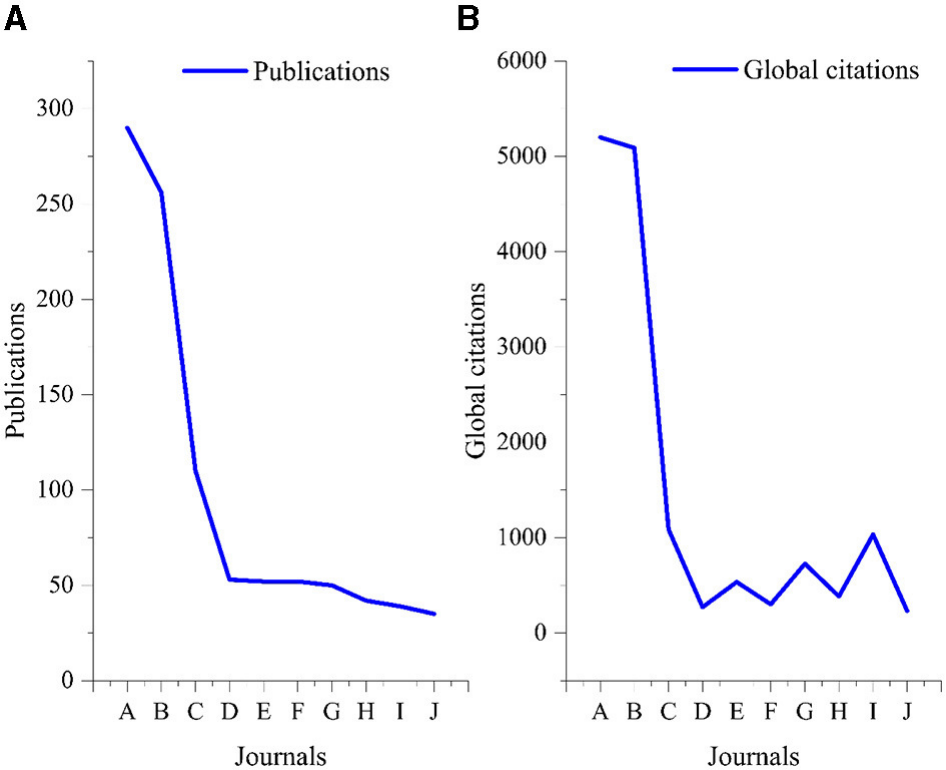
Top 10 most attractive journals in pediatric trauma research **(A)** Publications **(B)** Global citations. The Journal of Trauma and Acute Care Surgery (*n* = 124), previously known as The Journal of Trauma-Injury Infection and Critical Care (*n* = 166), after adding both values the Journal of Trauma and Acute Care Surgery. (https://journals.lww.com/jtrauma/pages/aboutthejournal.aspx) was ranked 1st (*n* = 290). A: The Journal of Trauma and Acute Care Surgery. B: Journal of Pediatric Surgery. C: Pediatric Emergency Care. D: Journal of the American College of Surgeons. E: Critical Care Medicine. F: Journal of Surgical Research. G: Annals of Emergency Medicine. H: American Surgeon. I: Pediatrics. J: Pediatric Surgery International.

The top three most prolific authors were Upperman JS (*n* = 29, citations = 202), Tepas JJ (*n* = 27, citations = 721), and Burd RS (*n* = 25, citations = 243) as shown in Figure 4. The most active funding agencies were the United States Department of Health Human Services (*n* = 156) and the National Institutes of Health (*n* = 124), as shown in Supplementary Figure 3. The most attractive journals in pediatric trauma research were *The Journal of Trauma and Acute Care Surgery* (*n* = 290, citations = 5,199) and the *Journal of Pediatric Surgery* (*n* = 256, citations = 5,088). Only three research journals published more than 100 papers on pediatric trauma, as shown in Figure 5. The most famous publishers in pediatric trauma research were Elsevier (*n* = 738) and Lippincott Williams & Wilkins (*n* = 697), as shown in Supplementary Figure 4.

**Figure 6:**
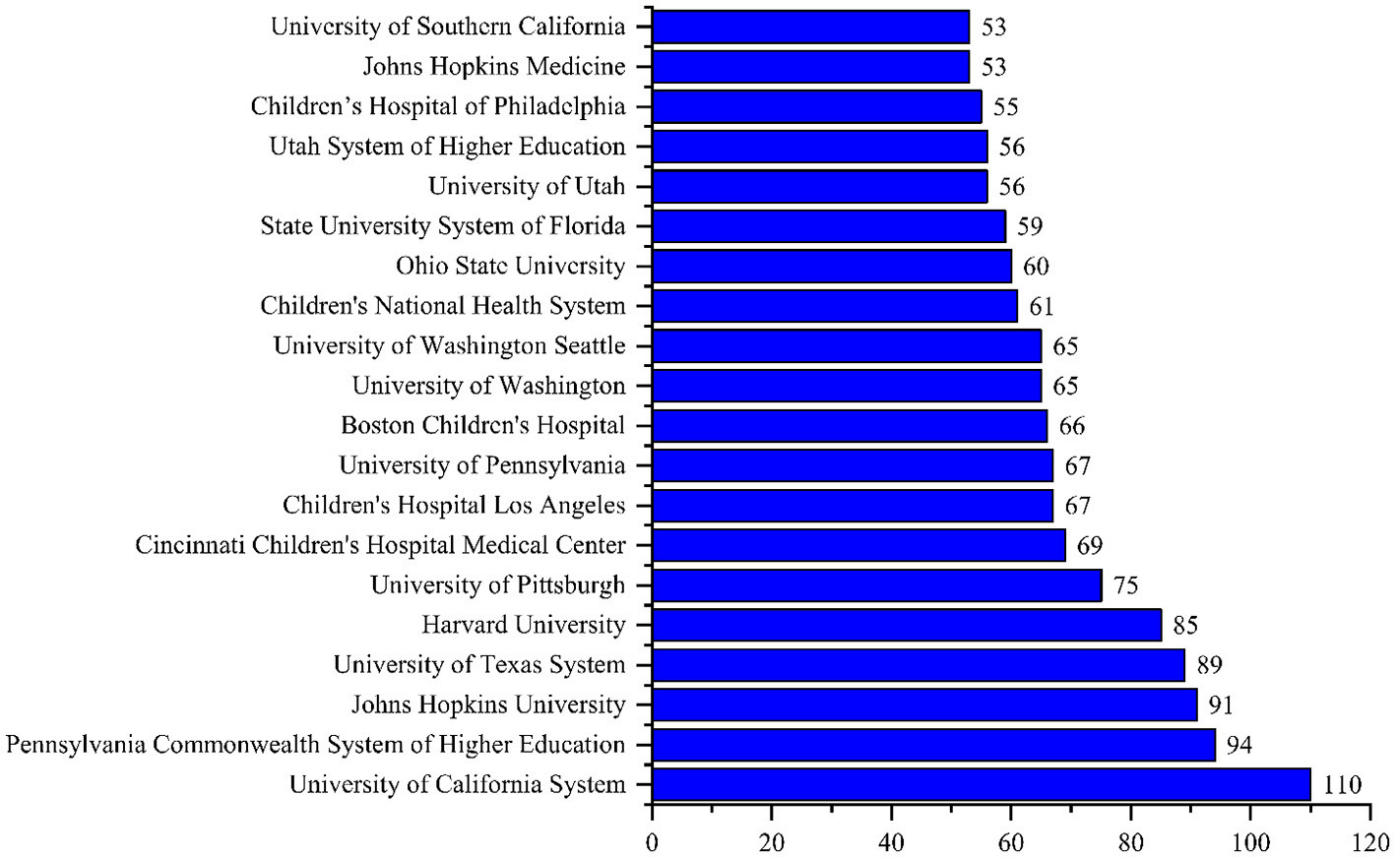
Top 20 most active institutes in pediatric trauma research.

**Figure 7:**
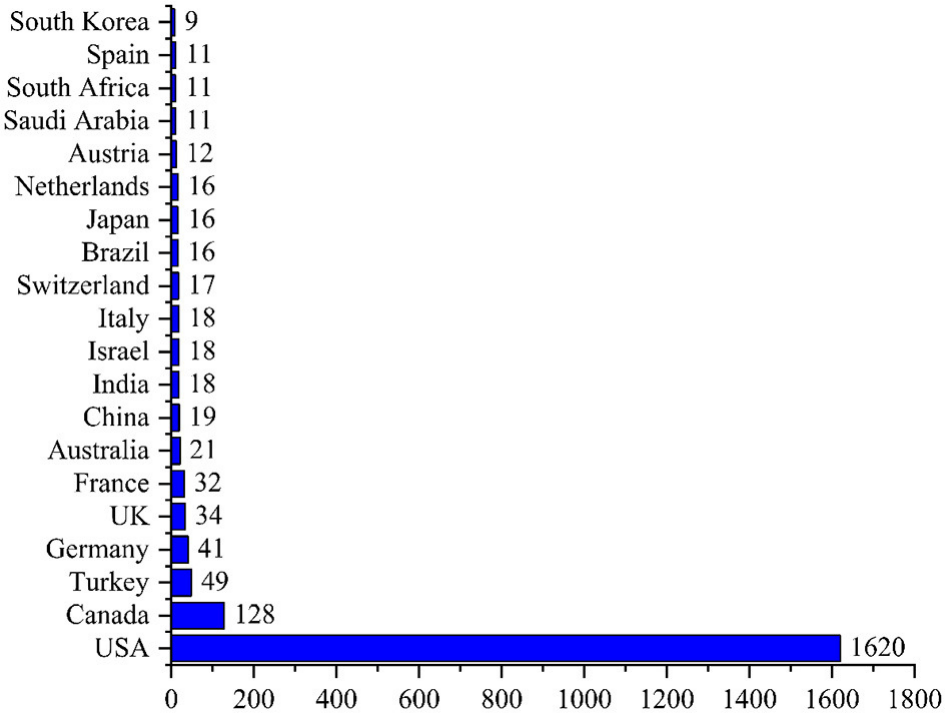
Top 20 most active countries in pediatric trauma research.

The top three most active institutes in pediatric trauma research were the University of California System (*n* = 110), Pennsylvania Commonwealth System of Higher Education (*n* = 94), and Johns Hopkins University (*n* = 91), as shown in Figure 6. The most dominant country was United States of America (*n* = 1,620, citations = 22,983), followed by Canada (*n* = 128, citations = 1,677), and Turkey (*n* = 49, citations = 244), as shown in Figure 7. The most cited paper in pediatric trauma was “Pediatric spinal trauma - review of 122 cases of the spinal cord and vertebral column injuries” published in the Journal of Neurosurgery (1988) cited 224 times (6.59 citations per year), as shown in Table 1.

**Table 1:** Top 10 most cited publications in pediatric trauma research.

**Ranking**	**Study reference**	**GCS**	**GCS/t**
1	Hadley MN, et al. Pediatric spinal trauma - review of 122 spinal cord and vertebral column injuries cases. *J Neurosurg*. (1988) 68:18–24.	224	6.59
2	Potoka DA, et al. Impact of pediatric trauma centers on mortality in a statewide system. *J Trauma-Inj Infect Crit Care*. (2000) 49:237–45.	219	9.95
3	Tepas JJ, et al. The pediatric trauma score as a predictor of injury severity in the injured child. *J Pediatr Surg*. (1987) 22:14–8.	201	5.74
4	Brown RL, et al. Cervical spine injuries in children: a review of 103 patients treated consecutively at a level 1 pediatric trauma center. *J Pediatr Surg*. (2001) 36:1107–14.	201	9.57
5	Imahara SD, et al. Patterns and outcomes of pediatric facial fractures in the United States: a survey of the National Trauma Data Bank. *J Am College Surg*. (2008) 207:710–6.	144	10.29
6	Blackwell CD, et al. Pediatric head trauma: changes in use of computed tomography in emergency departments in the United States over time. *Ann Emerg Med*. (2007) 49:320–4.	142	9.47
7	Hall JR, et al. The outcome for children with blunt trauma is best at a pediatric trauma center. *J Pediatr Surg*. (1996) 31:72–7.	137	5.27
8	Lustrin ES, et al. Pediatric cervical spine: normal anatomy, variants, and trauma. *Radiographics*. (2003) 23:539–60.	134	7.05
9	Mcgraw BL and Cole RR. Pediatric maxillofacial trauma - age-related variations in injury. *Arch Otolaryngol-Head Neck Surg*. (1990) 116:41–5.	130	4.06
10	Mayer T, et al. The modified injury severity scale in pediatric multiple trauma patients. *J Pediatr Surg*. (1980) 15:719–26.	126	3.00

*GCS, Global citation score; GCS/t, Global citation score per year*.

### Network Visualizations

#### Co-authorship Countries Network Visualization

**Figure 8:**
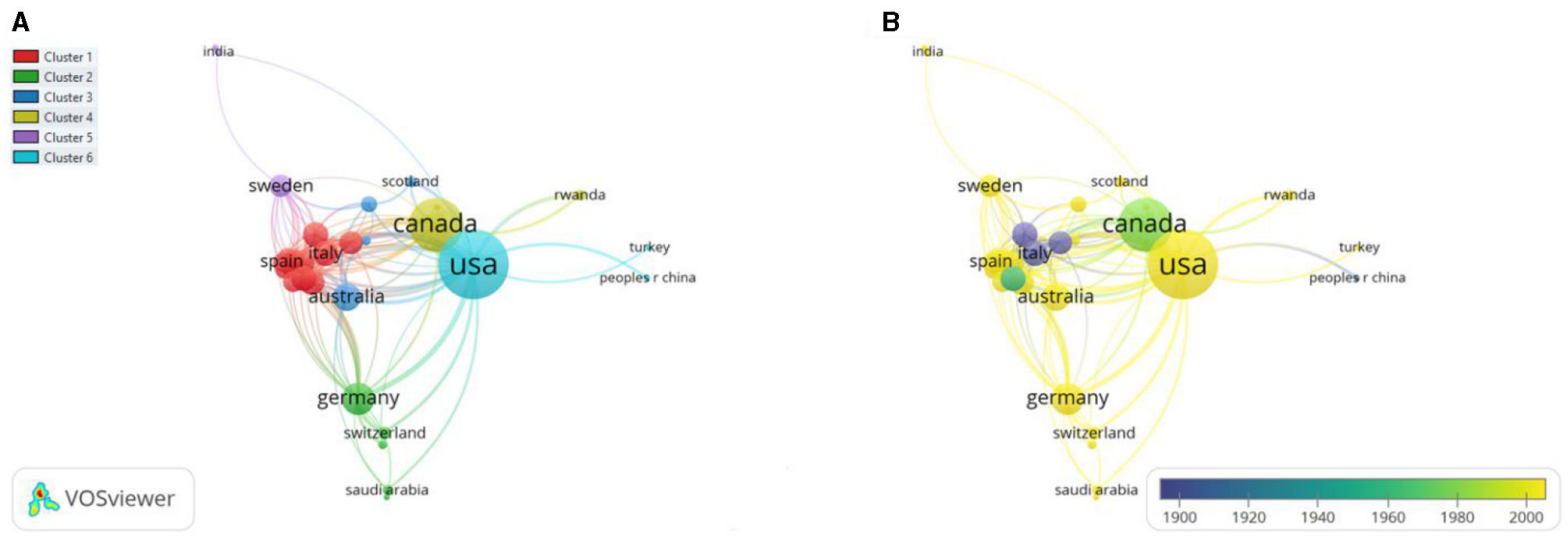
Co-authorship countries **(A)** Network visualization. **(B)** Overlay visualization by time (year).

The minimum number of documents of a country was fixed at three of the total countries, only 34 countries met the threshold and were plotted. Based on total link strength (TLS), the top-3 countries were the United States of America (TLS = 103), Canada (TLS = 70), and Germany (TLS = 34). The co-authorship country network visualization is presented in Figure 8.

#### Co-authorship Organizations Network Visualization

**Figure 9:**
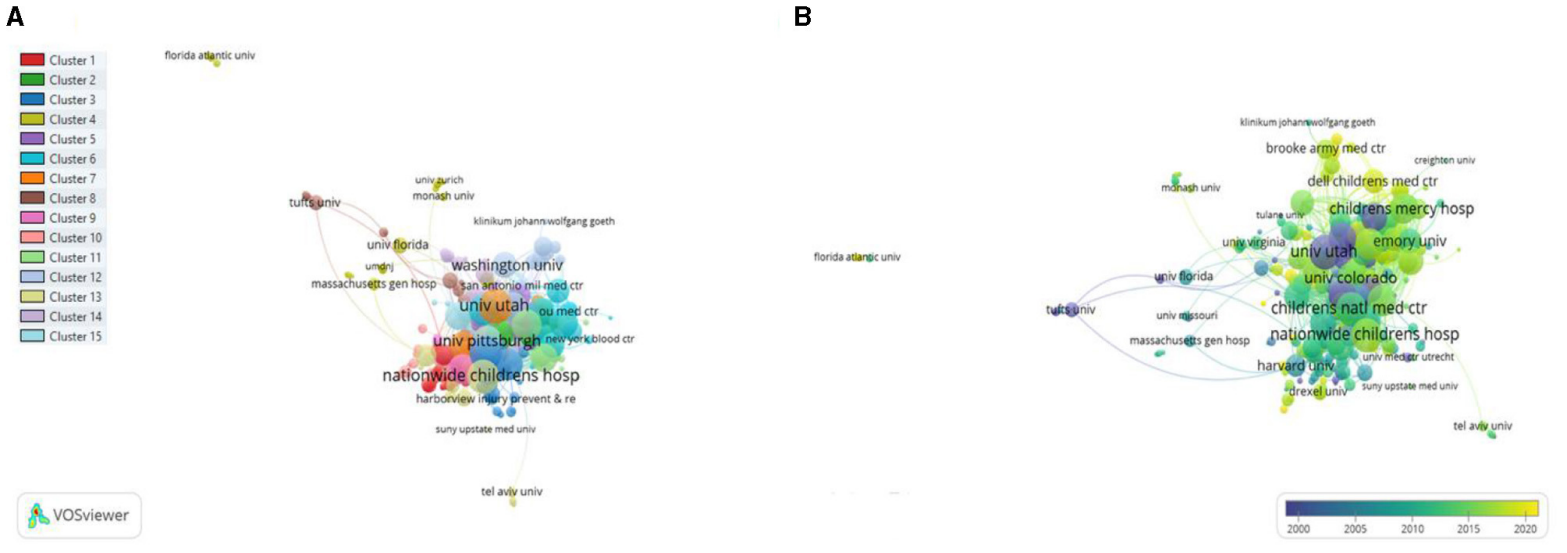
Co-authorship organizations **(A)** Network visualization. **(B)** Overlay visualization by time (year).

The minimum number of documents of an organization was fixed at three. A total of 318 organizations were plotted. The minimum number of cluster items were fixed at 10, a total of 15 clusters were formed. The University of Utah had the highest TLS = 132, followed by Nationwide Children's Hospital (TLS = 130), and Children's National Medical Center (TLS = 126), as shown in Figure 9.

#### Co-occurrence Author Keywords

**Figure 10:**
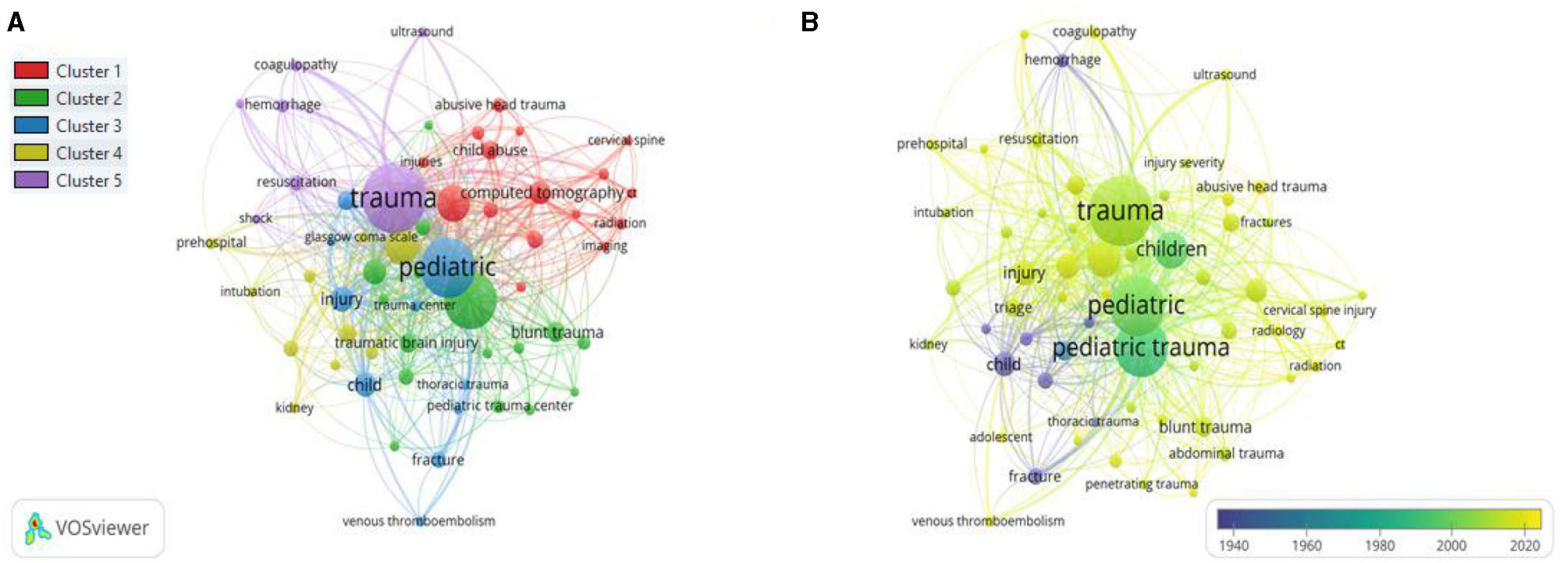
**(A)** Author keywords network visualization. **(B)** Overlay visualization by time (year).

The minimum number of keywords occurrences was fixed at 10. Of the total keywords, only 59 keywords were plotted. The top three author keywords were trauma (TLS = 513), pediatric (TLS = 417), and children (TLS = 176). The co-occurrence author keywords network visualization is presented in Figure 10.

## Discussion

The bibliometric properties of 2,269 documents incorporated in the citation indexes of pediatric trauma studies conducted in the last 53 years were examined. Bibliometric analysis plays a significant role by providing the referral point to the researchers, policymakers, and practitioners. The current study focused on the global research trends and outcomes on pediatric trauma.

Pediatric and trauma been identified as an important keywords. The most frequently used other keywords were patients, blunt, injury, center, head, management, and care, respectively. It can be used by researchers for accessing studies in this field. The most studied research areas in pediatric trauma were surgery, followed by pediatrics, general internal medicine, emergency medicine, and neurosciences neurology.

When the network visualization map for international cooperation is examined, the country with a significantly higher number of articles published on pediatric trauma is the USA. The current study has similar trends with many other bibliometric studies in different fields that confirm the USA as a global research leader both quantitative and qualitatively (17–22). This leading position may be due to economic strength that leads them to spare ample funds to carry out research in important research areas, emerging technology, and public health issues. Moreover, accessibility to research facilities and active collaboration with other local and international institutes results in better publications and citation frequency. Contrary to other studies (23), China is not listed among the top leading countries in terms of publication and citations on pediatric trauma. This may be due to negligence in this research area, or the incidences may be very low and have not gotten researchers' attention.

The most prolific authors in pediatric trauma studies and the global citations to their work differed. The most prolific author was Upperman JS, while the most cited author was Tepas JJ. The citation rates of the studies that determine the requirements related to the subject and direct the field is high (24).

Journals are considered important tools for the dissemination of research; thus, quality and prestige of a journal play a major role in transmitting the research to the concerned segment of society (25). Our results showed that these papers have been published in three main journals; *The Journal of Trauma and Acute Care Surgery*; *Journal of Pediatric Surgery*; and *Pediatric Emergency Care*. The global citation score of papers published in these journals were also high. Authors that intend to have highly-cited papers can publish in these journals. In total, 63.24% documents were published in Elsevier and Lippincott William and Wilkins hosted journals. In term of open access, about 17% published documents in pediatric trauma had all open access services and available free of cost for academic and research purposes. Funding agencies and research organizations' role in the promotion of science and research is of key importance (26). The results show that most of the funding agencies were from the USA and other developed countries. The finding of our study is in line with other studies (27). Similarly, the institutes and hospitals publishing the high cited publications and higher number of publications have a similar trend as that of funding. This is evident that the funding has a direct relation with the scientific publication. Thus, the countries with limited resources usually cannot compete with the resource-rich countries.

This study will provide a reference point to the researcher and practitioners and be a baseline to devise policy to effectively manage pediatric trauma. Moreover, there is a dire need for research collaboration and exchange of skills with scientists from low-income countries to effectively equip them with facilities to deal with pediatric trauma.

### Study Limitations and Strength

In this study, only one database was used for data retrieval. The search queries were limited to the title filed, which could be another limitation. The main strength of the current study is the use of bibliometric and citations tools to evaluate some key bibliometric indicators and network visualization mapping of the scientific publications on pediatric trauma.

## Conclusion

In recent years, bibliometric type studies have gained significant attention not only providing a comprehensive overview of the published literature but also identifying the missing gaps, research frontiers and future research trends. The current study provides a comprehensive overview of global research outcomes and useful insights in the field of pediatric trauma. In the past few decades, studies on pediatric trauma have been increased. The most productive country was USA participated in more than 70% of publications on pediatric trauma, while the leading institution was the University of California System. The most attractive journal in pediatric trauma was *The Journal of Trauma and Acute Care Surgery*. This study might be helpful for the scientific community and academia to understand the recent achievements and future research trends in the field of pediatric trauma. Furthermore, collaborative research initiative needs to be established between institutions of developing countries with developed nations.

## Data Availability Statement

Publicly available datasets were analyzed in this study. All the data is provided in the article. Further data can be provided on request.

## Ethics Statement

Ethical review and approval was not required for the study on human participants in accordance with the local legislation and institutional requirements. Written informed consent from the participants' legal guardian/next of kin was not required to participate in this study in accordance with the national legislation and the institutional requirements.

## Author Contributions

TA: designed this study, conducted the initial search, collected the data, performed all the analyses and generated the graphs, and wrote the first draft. TA, LH, MK, GN, SK, and H: helped in manuscript writing and editing. TA, İÇ, SJ, MB, HJ, and XW: review and proofreading. All authors contributed to the article and approved the submitted version.

## Funding

This study was funded by Henan Province science and technology attack plan project (212102310044), Hubei Provincial Health Commission Joint Fund Project from 2019 to 2021 (WJ2019H299). The value of brainstem auditory evoked potentials in the very early diagnosis of childhood autism number: WJ2019H299.

## Conflict of Interest

The authors declare that the research was conducted in the absence of any commercial or financial relationships that could be construed as a potential conflict of interest.

## Publisher's Note

All claims expressed in this article are solely those of the authors and do not necessarily represent those of their affiliated organizations, or those of the publisher, the editors and the reviewers. Any product that may be evaluated in this article, or claim that may be made by its manufacturer, is not guaranteed or endorsed by the publisher.
