# Supplementary material for: Global Research Trends in Pediatric Trauma From 1968 to 2021: A Bibliometric Analysis
Source: Front Pediatr. 2021 Oct 28;9:762531. doi: 10.3389/fped.2021.762531 (PMC8581173; doi:10.3389/fped.2021.762531)
Supplement: Supplementary file 1 [file Data_Sheet_1.docx]

**Supplementary Figure 1.** Publication language of included documents

**Supplementary Figure 2.** Document types of included publications

**Supplementary Figure 3.** Top 5 funding agencies in pediatric trauma research

**Supplementary Figure 4.** Top 10 famous publishers in pediatric trauma research
